# Transition metal modified cordierite for anthracene removal and low-cost exhaust microcontaminant control

**DOI:** 10.1038/s41598-025-25662-5

**Published:** 2025-11-25

**Authors:** Wiktor Pacura, Jerzy Górecki, Estelle Marie M. Vanhaecke, Katarzyna Szramowiat-Sala, Małgorzata Gierek, Janusz Gołaś

**Affiliations:** 1 The Department of Renewable Energy and Environmental Research, Division of Bioenergy Conversion Technology, Mineral and Energy Economy Research Institute of the Polish Academy of Sciences (MEERI PAS), Krakow, Poland; 2 Faculty of Energy and Fuels, AGH University of Krakow, Krakow, Poland; 3 Department of Chemical Engineering, Faculty of Natural Sciences, Norwegian University of Science and Technology, Trondheim, Norway

**Keywords:** Vehicle exhaust emissions, Air micro-contaminants, Polycyclic aromatic hydrocarbons, Anthracene removal, Cordierite-based materials, Transition metal modification, Atmospheric chemistry, Environmental impact, Chemical engineering

## Abstract

Unregulated micro-contaminants such as polycyclic aromatic hydrocarbons (PAHs) from gasoline vehicles pose increasing environmental and health risks. In this study, cordierite granules were thermally pretreated, acid-activated and modified with transition metal oxides (CuO, Fe₂O₃, MnO₂) by wet impregnation and calcination at 600 °C, producing well-defined oxide coatings confirmed by TGA/DSC and SEM/EDS analyses. A custom-built test rig introduced anthracene vapor (200 µg at 300 °C, 60 mL min⁻¹) through a fixed bed of the modified cordierite, and the downstream Tenax trap was analyzed by GC-MS to quantify removal efficiency. Surface characterization revealed uniform CuO and Fe₂O₃ distributions but agglomerated MnO₂ with residual chlorine, correlating strongly with catalytic performance. Copper- and iron-modified cordierite achieved up to 25.3% and 18.2% net anthracene removal respectively, while MnO₂ was markedly less effective. These results demonstrate that low-cost, non-noble metal coatings can enhance PAH capture and partial oxidation in exhaust-like conditions and provide a mechanistic basis for developing sustainable emission control materials. These findings highlight the potential of non-noble metal oxide coatings for enhancing PAH mitigation in exhaust systems and provide a foundation for future application-oriented development of sustainable emission control materials.

## Introduction

 The global vehicle fleet exceeds 1.3 billion units, with over 250 million registered in the European Union alone^1,2^. In 2019, passenger vehicles traveled over 2.8 trillion kilometers in the EU, underscoring the scale of mobile emission sources^3,4^. As a response, stringent emission regulations – such as Euro standards and equivalents in China, Japan, and North America – have been introduced, targeting carbon monoxide (CO), carbon dioxide (CO₂), nitrogen oxides (NOₓ), total hydrocarbons (THC), and particulate matter (PM/PN)^5–7^. Some jurisdictions enforce even stricter local limits^8^, particularly for CO₂.

However, beyond regulated pollutants, internal combustion engines emit a variety of unregulated micro-contaminants originating from incomplete combustion, lubricant degradation, and material wear^4^. Among these, polycyclic aromatic hydrocarbons are of major concern due to their carcinogenic and mutagenic properties^9^. Although present at trace concentrations, PAHs pose a significant health risk owing to their persistence^9,10^, bioaccumulation potential, and low-stack emission profile – placing them close to the breathing zone in urban environments^11,12^.

Gasoline vehicles are typically equipped with after-treatment systems such as three-way catalysts (TWCs), exhaust gas recirculation (EGR), and gasoline particulate filters (GPFs). TWCs utilize platinum group metals (PGMs) to oxidize CO and hydrocarbons and reduce NO_x_ to N₂, while GPFs – often made from cordierite – trap particulate matter via physical filtration^13^. In some cases, GPFs are coated with PGMs or ceria to function as “four-way catalysts”^14,15^. While these systems are effective for regulated pollutants, their efficiency in mitigating PAHs remains poorly characterized.

Recent research has increasingly focused on the control of unregulated exhaust micro-contaminants such as PAHs, VOCs and semi-volatile species that are not efficiently captured by conventional three-way catalysts or particulate filters. Studies have demonstrated that gasoline particulate filters based on cordierite substrates can effectively reduce particle-bound PAHs but show variable performance for vapor-phase PAHs, suggesting that additional sorptive or catalytic functionalities are required^16–18^. Various catalytic coatings have been proposed to enhance micro-contaminant removal, including platinum-group metals, ceria–zirconia mixtures and transition metal oxides^14,15^. However, the high cost and supply risk of PGMs have motivated exploration of low-cost alternatives^19^.

Cordierite, widely used as a ceramic substrate for catalytic converters and GPFs due to its thermal stability and low backpressure, has also been investigated as a support for non-noble metal coatings. Transition metal oxides such as Fe₂O₃, CuO, MnO₂ and Co₃O₄ have shown promising activity in degrading PAHs and VOCs in incineration flue gas, soils and aqueous systems^20–22^. Yet, few studies have systematically tested such coatings under conditions representative of gasoline exhaust streams – characterized by short residence times and fluctuating thermal profiles. Moreover, the mechanistic relationship between oxide dispersion, redox properties and PAH capture on cordierite remains underexplored.

This study aims to evaluate the potential of cordierite modified with selected transition metals (Fe, Cu, Mn) to remove anthracene from an airstream under simulated gasoline exhaust conditions. The influence of metal type and loading (1%, 3%, 5%) on removal efficiency is assessed using a custom-designed laboratory setup. The work also includes surface and thermal characterization of the modified materials to understand structure–activity relationships and identify limitations for practical implementation. The findings contribute to the development of non-PGM alternatives for micro-contaminant control in vehicle emissions and provide preliminary insights into the feasibility of transition-metal-modified ceramic supports for future exhaust after-treatment systems.

## Materials and methods

### Materials and reagents

Cordierite (type C410) was selected as the support material due to its thermal stability, low porosity, and inert chemical nature. The differences in chemical composition between porous type C520 and C410 of cordierite is presented in Table 1. Transition metal precursors – iron(III) nitrate nonahydrate (Fe(NO₃)₃·9 H₂O), copper(II) nitrate trihydrate (Cu(NO₃)₂·3 H₂O), and manganese(II) chloride tetrahydrate (MnCl₂·4 H₂O) – were used for material modification. All reagents were of analytical grade.

**Table 1:** Chemical composition of type C410 and type C520 Cordierite.

Type	SiO_2_	Al_2_O_3_	Fe_2_O_3_	MgO	K_2_O + Na_2_O	CaO	BaO	TiO_2_
C410 C520	50.80 52.99	33.45 30.93	0.64 2.21	6.91 3.42	2.11 0.62	0.22 6.54	0.10 –	0.35 –

The values do not add to 100% due to approximation. Data based on provided certificates by producers

Anthracene (97%, Sigma-Aldrich) was selected as a representative PAH due to its typical occurrence in gasoline engine emissions, low toxicity, and suitable volatility. Anthracene-d₁₀ was used as the internal standard for GC-MS quantification.

### Material preparation

Raw cordierite granules (type C410) were subjected to a thermal pretreatment to ensure material stability and reproducibility. The samples were placed in ceramic crucibles and sintered in a laboratory muffle furnace at 1100 °C for 1 h, using a heating rate of 200 °C/h. After sintering, the furnace was left to cool overnight, and the cordierite was stored in sealed containers to prevent moisture uptake.

To assess the effect of surface activation, the sintered cordierite was treated with concentrated nitric acid. The procedure involved refluxing the material in 26% HNO₃ (10 mL per gram of material) at ~ 105 °C for 24 h using a round-bottom flask equipped with a water-cooled condenser. Post-treatment, the samples were rinsed with deionized water, dried at 105 °C, and stored in air-tight containers.

Transition metal modification was performed via wet impregnation. Aqueous solutions of iron(III) nitrate nonahydrate (Fe(NO₃)₃·9 H₂O), copper(II) nitrate trihydrate (Cu(NO₃)₂·3 H₂O), and manganese(II) chloride tetrahydrate (MnCl₂·4 H₂O) were prepared to achieve metal loadings of 1%, 3%, and 5% by mass relative to the cordierite. Each impregnation was performed by adding 5 g of cordierite to 25 mL of metal salt solution in a 200 mL Erlenmeyer flask. The mixture was shaken horizontally for 10 min to ensure homogeneous distribution, then dried at 105 °C in a laboratory oven, with manual mixing every 10–15 min to prevent precipitation and layering.

The dried samples were subsequently calcined in a muffle furnace. The temperature was ramped at 150 °C/h to a final temperature of 600 °C, which was held for 4 h. The selection of this temperature was based on thermogravimetric analysis (TGA), confirming complete decomposition of nitrate and chloride precursors and formation of respective metal oxides – CuO, Fe₂O₃, and MnO₂.

**Fig. 1:**
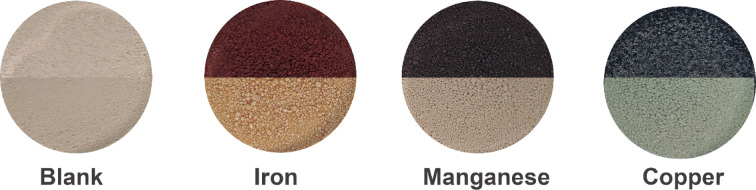
Colour change of the material. Top row show material after calcination, bottom before. From left: unmodified blank, 5% iron; 5% manganese; 5% copper. Difference in colour of blank is due to changes in light source. (source: own elaboration).

Figure 1 shows the visual transformation of the materials before and after calcination. Noticeable color changes confirm the formation of metal oxides: copper-impregnated samples turned dark blue, iron-treated samples exhibited a reddish-brown hue, and manganese samples became black.

### Thermogravimetric analysis

Thermogravimetric analysis combined with differential scanning calorimetry (DSC) analysis was performed using a Netzsch STA449 F3 Jupiter system under a synthetic air/argon flow to determine the optimal calcination temperature. The heating rate was 10 °C/min up to 800 °C, with an isothermal hold at 800 °C for 10 min. During analysis flow of gases was set as following: argon 25 ml / min, synthetic air N_2_/O_2_ (20:80%_vol_.) 55 ml/min. Data confirmed multistage decomposition of metal salts and supported oxide formation.

### Surface and morphological characterization

Surface morphology and elemental composition were assessed using a field emission scanning electron microscope (FE-SEM, FEI Versa 3D) equipped with energy-dispersive X-ray spectroscopy (EDS). The SEM configuration used was consistent with that of previous studies^7,13,18^. Analyses were conducted in high vacuum using 5 kV for imaging and 20 kV for EDS measurements. SEM images were acquired in backscattered electron (BSE/CBS) mode to enhance the contrast of metal oxide coatings against the cordierite substrate. No conductive coating was applied. To ensure efficient generation of X-rays, the overvoltage had to be no less than twice the critical ionization energy of the elements of interest^23^.

### Anthracene removal setup

**Fig. 2:**
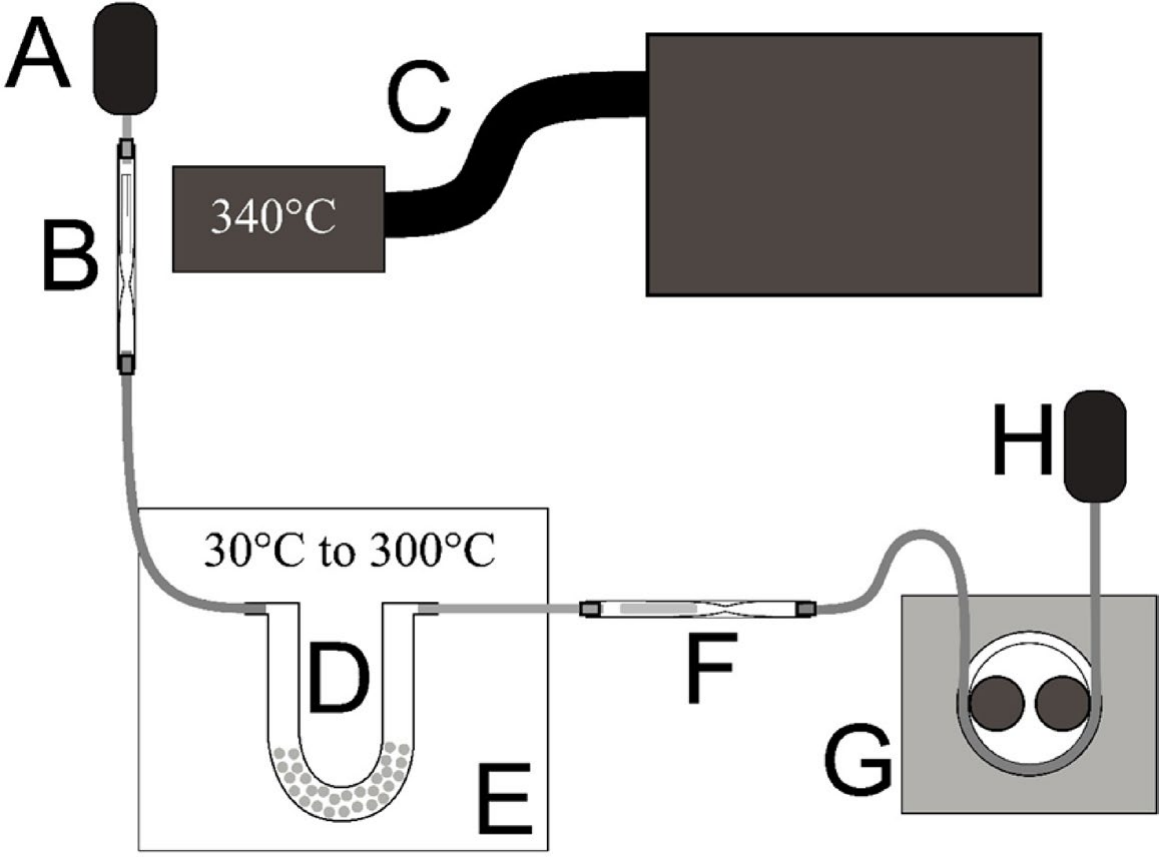
Schematic of the anthracene removal test setup. (**A**) Pressurized air source; (**B**) Anthracene evaporation unit with external heating; (**C**) Glass injector tube containing aluminum foil with anthracene; (**D**) U-tube filled with 10 g of test material; (**E**) Laboratory oven; (**F**) Tenax TA 60/80 trap for capturing unreacted anthracene; (**G**) Peristaltic pump regulating flow; (**H**) Vapor outlet. The U-tube was placed horizontally to ensure uniform gas–solid contact and avoid channeling. The entire system was assembled using chemically inert PTFE connectors and tubes. (source: own elaboration)

To simulate real-world emission of PAHs from gasoline vehicle exhaust, a laboratory-scale test rig (Fig. 2) was designed to assess anthracene removal by modified cordierite under controlled thermal and flow conditions.

A solution of anthracene (200 µg dissolved in 1 cm³ of cyclohexane) was pipetted onto a piece of aluminum foil. This foil was carefully rolled and inserted into a glass evaporation tube, which was externally heated and maintained at 340 °C to ensure complete vaporization of anthracene. The vaporized anthracene was then introduced into the system by a peristaltic pump operating at a flow rate of 60 mL/min. The gas stream passed through PTFE tubing (inner diameter: 4 mm) into a U-shaped glass tube (U-tube) filled with 10 g of test material – either unmodified (blank) cordierite or transition-metal-modified samples.

The U-tube was placed horizontally inside an electric oven, which was programmed to ramp from ambient temperature to 300 °C at a heating rate of 35 °C/min, then hold this temperature for 27 min, totaling 35 min per run. The temperature was selected to reflect the exhaust thermal window of gasoline engines equipped with GPF systems.

Downstream of the test material, a secondary glass tube filled with 0.15 g of Tenax TA 60/80 sorbent (secured by quartz wool) acted as the anthracene trap. This trap was located outside the oven to ensure ambient conditions and prevent further thermal degradation or slippage of anthracene vapor. After each run, the trap was immediately sealed to avoid post-experimental losses or contamination.

Control experiments were conducted using:

- An empty U-tube (no material) to assess baseline transport and breakthrough,
- Unmodified cordierite to evaluate the material’s intrinsic sorption capacity,
- A multistage cold-trap and wash-bottle configuration (see Supplementary Materials) to validate the completeness of anthracene capture.

All glassware was rinsed post-experiment with cyclohexane to recover any potential anthracene residues deposited on internal surfaces.

### Sample extraction and GC-MS analysis

Following each experimental run, the Tenax TA 60/80 sorbent and the two quartz wool plugs were carefully transferred into a clean 10 mL glass vial. To ensure complete recovery of adsorbed anthracene, the original glass tube was rinsed with 4 cm³ of high-purity cyclohexane (Chempur, 99.5%), which was then used to cover the sorbent and wool in the vial. The vial was sealed with a PTFE-lined cap and subjected to ultrasonic extraction at 70 °C for 30 min to desorb the analyte into the solvent phase.

After cooling to room temperature, the extract was filtered (if needed) and transferred to a GC-MS vial for analysis. One milliliter of each extract was analyzed using a ThermoScientific Trace 1310 gas chromatograph coupled to an ITQ 900 ion trap mass spectrometer. Autosampling was performed with a TriPlus RSH system.

Separation of PAHs was achieved using a DB-5MS UI capillary column (25 m × 0.25 mm i.d. × 0.25 μm film thickness) with a stationary phase consisting of 5% diphenyl/95% dimethylpolysiloxane. The column temperature program was as follows:

- Initial temperature: 65 °C (held for 2 min),
- Ramp 1: 20 °C/min to 150 °C (hold 4 min),
- Ramp 2: 10 °C/min to 250 °C (no hold),
- Ramp 3: 20 °C/min to 300 °C (hold 6 min).

The injector and transfer line temperatures were maintained at 310 °C. The ion trap temperature was set to 250 °C. Helium (6.0 purity) was used as the carrier gas at a constant flow rate of 1.2 mL/min. A triple gas cartridge filter (ThermoScientific) was installed upstream to remove residual oxygen, moisture, and hydrocarbons from the carrier gas.

Quantification of anthracene was performed using external calibration curves prepared freshly for each analysis batch. Calibration standards were prepared by serial dilution of stock solutions of anthracene and the internal standard anthracene-d₁₀ (Sigma-Aldrich, 98%) in cyclohexane. Detection was based on selective ion monitoring (SIM) of characteristic ions:

- Anthracene: quantifier ion 178 m/z; qualifiers 176 and 179 m/z,
- Anthracene-d₁₀: quantifier ion 188 m/z; qualifiers 187 and 189 m/z.

Retention times were consistent at ~ 16.92 min for both compounds under the applied conditions. Identification was confirmed by both retention time and ion ratios, and quantitation was based on the response ratio of analyte to internal standard.

All sample batches were analyzed in triplicate to ensure repeatability. The GC–MS quantification exhibited a < 3% margin of error (standard deviation below 1.5 ppm). Before calculating mean removal efficiencies, raw data were screened for outliers using the Q-Dixon test (*p* < 0.05). Blank samples (solvent only, unmodified cordierite) were analyzed regularly to monitor for contamination and carryover.

### Calculation of removal efficiency

Anthracene removal efficiency was determined by comparing the amount of anthracene detected in the Tenax trap after each experiment to the known initial mass introduced into the system (200 µg). Quantitative data from the GC-MS analysis were used to calculate both absolute and relative removal values.

Three key metrics were computed for each sample:

- Total anthracene removed (uncorrected) – calculated as the difference between the source amount and the amount recovered from the trap (Eq. 1):

1 $$A_{{removed,uncorrected}} = A_{{source}} - A_{{trap}}$$

b. Anthracene removal due to modification only – calculated by subtracting the removal efficiency of the unmodified cordierite (blank) from that of the modified material (Eq. 2):

2 $$\:{A}_{removed,\:net}=\:{A}_{removed,\:uncorected}-\:{A}_{removed,blank}$$

c. Removal efficiency [%] – calculated as (Eq. 3):

3 $$\:\eta\:=\:\left(\frac{{A}_{removed,net}}{{A}_{source}}\right)\times\:100$$

Where:

- $\:{A}_{source}$ is the mass of anthracene introduced (200 µg),
- $\:{A}_{trap}$ is the mass detected in the Tenax sorbent,
- $\:{A}_{removed,blank}$ is the amount removed by unmodified cordierite (baseline sorption).
- η is the net removal efficiency expressed as a percentage.

The blank sample consistently removed ~ 17.8 µg of anthracene (~ 9%), which was subtracted from total removal values of the modified materials to isolate the effect of the transition metal coating.

To ensure data accuracy:

- All measurements were conducted in triplicate,
- Control runs with empty tubes confirmed system tightness and negligible losses due to condensation or adsorption on walls.

This methodology ensured that calculated removal efficiencies reflect the catalytic or sorptive effect of the metal-modified surface rather than passive trapping or system artifacts.

## Results and discussion

### Thermal decomposition and oxide formation

Thermogravimetric analysis was employed to investigate the thermal stability and decomposition behavior of the metal salt precursors used in the wet impregnation process. This analysis was critical for determining the appropriate calcination temperature required to fully convert the metal precursors into their corresponding oxides without leaving residual anions (nitrate or chloride), which could interfere with surface activity or lead to undesirable byproducts during potential application in emission systems.

For copper nitrate (Cu(NO₃)₂·3 H₂O), the TGA curve showed a multi-stage mass loss profile. The initial mass loss (~ 25–150 °C) was attributed to the removal of crystalline and adsorbed water. The second, more pronounced loss (~ 200–280 °C), was associated with the decomposition of nitrate groups, releasing NOx gases and forming CuO as the final oxide product. Literature confirms^24^ that Cu(NO₃)₂ undergoes decomposition in air via the simplified reaction pathway (4):

4 $$\:{Cu\left(N{O}_{3}\right)}_{2}\cdot\:3{H}_{2}O\:\to\:CuO+2N{O}_{2}+\frac{1}{2}{O}_{2}+3{H}_{2}O$$

**Fig. 3:**
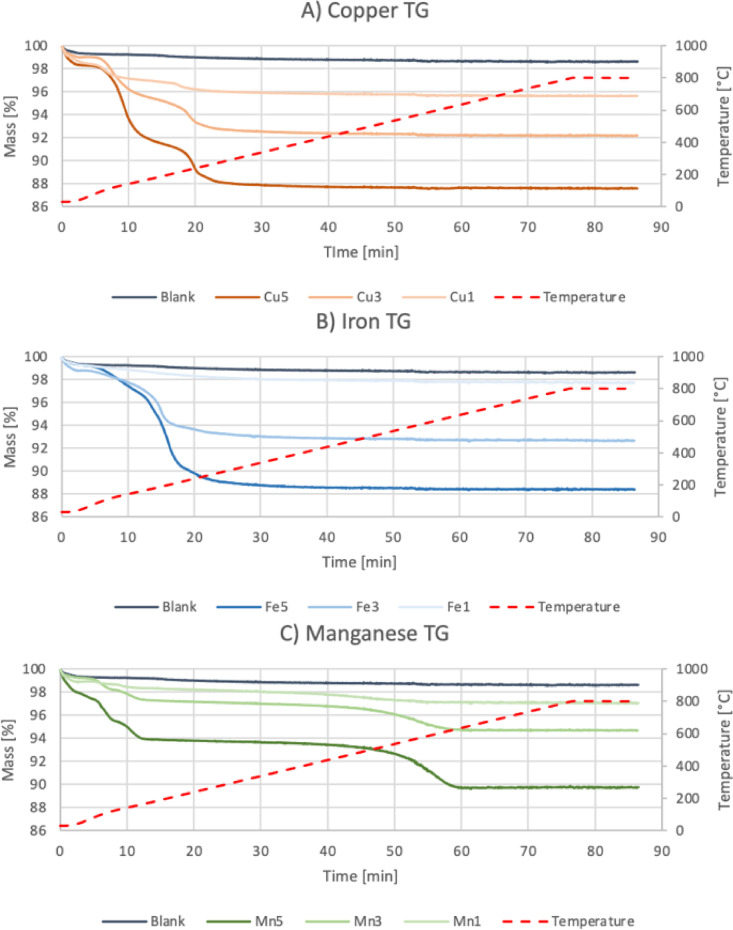
Thermogravimetry graphs of modified materials. Graph A shows cordierite modified with copper; graph B iron; graph C manganese. On right axis, the mass loss is presented. Left axis corresponds to the temperature, which is presented as red line.

The final residual mass plateaued near 300 °C, confirming the completion of thermal decomposition (Fig. 3A).

For iron nitrate (Fe(NO₃)₃·9 H₂O), a similar two-step decomposition behavior was observed. Initial dehydration occurred below 150 °C, followed by exothermic decomposition of nitrate ligands between 200 °C and 300 °C. This transformation is accompanied by the release of nitrogen oxides and water vapor, with Fe₂O₃ identified as the final solid product. The general decomposition described by Wieczorek-Ciurowa and Kozak, 1999^25^ can be expressed as in the following reaction (5):

5 $$\:2Fe{\left(N{O}_{3}\right)}_{3}\cdot\:9{H}_{2}O\:\to\:\:{Fe}_{2}{O}_{3}+6NO+\:\frac{3}{2}{O}_{2}+3{H}_{2}O$$

This process was completed around 300 °C, with stable weight observed up to 600 °C (Fig. 3B).

In contrast, manganese chloride (MnCl₂·4 H₂O) exhibited a more complex thermal behavior. The TGA curve showed a first stage mass loss below 250 °C, attributed to the stepwise release of hydration water^26,27^. Unlike nitrate salts, MnCl₂ is more thermally stable, with decomposition of the anhydrous salt initiating only above ~ 400 °C^27^. The final mass loss occurred between 400 and 600 °C, where oxidation of Mn²⁺ in air leads to the formation of MnO₂ or mixed valence oxides such as Mn₂O₃^28^, depending on the oxygen partial pressure and duration of heating (6):

6 $$\:MnC{l}_{2}+\frac{1}{2}{O}_{2}\to\:Mn{O}_{2}+{Cl}_{2}$$

MnCl₂ was selected as the manganese source due to its high solubility, high purity and commercial availability at a reasonable price. The use of a chloride precursor was driven mainly by practical considerations during the experimental planning phase. However, residual chlorine species observed in EDS results (Sect. 3.2) suggest incomplete conversion, possibly due to kinetic limitations or insufficient oxidation conditions during calcination (Fig. 3C), which is consistent with the multistep decomposition behavior of MnCl₂ reported by Yi et al.^26^ and the sensitivity of MnO_x_ phase formation to precursor choice and oxygen partial pressure^29^. Such residual Cl may interfere with oxide dispersion and active site availability, thereby reducing catalytic efficiency.

Based on these findings, 600 °C was selected as a universal calcination temperature for all samples. This temperature ensures:

- Complete dehydration and decomposition of metal salts,
- Full formation of oxide phases (CuO, Fe₂O₃, MnO₂),
- Thermal stability of the cordierite substrate,
- And consistency across metal-modified materials.

While higher temperatures may enhance crystallinity, they could also promote oxide sintering or reduce surface area – undesirable effects for catalytic or sorptive performance^30^. Hence, 600 °C was identified as a balanced compromise between thermal conversion and structural preservation. These thermogravimetric results provide essential insight into the nature of active species formed during modification and lay the foundation for interpreting performance trends discussed in later sections.

### Morphology and surface composition

The surface morphology and elemental distribution of the modified cordierite samples were investigated using scanning electron microscopy coupled with energy-dispersive X-ray spectroscopy. The analysis aimed to assess the homogeneity of metal oxide distribution, surface texture changes induced by acid treatment and calcination, and the relationship between metal loading and surface composition.

**Fig. 4:**
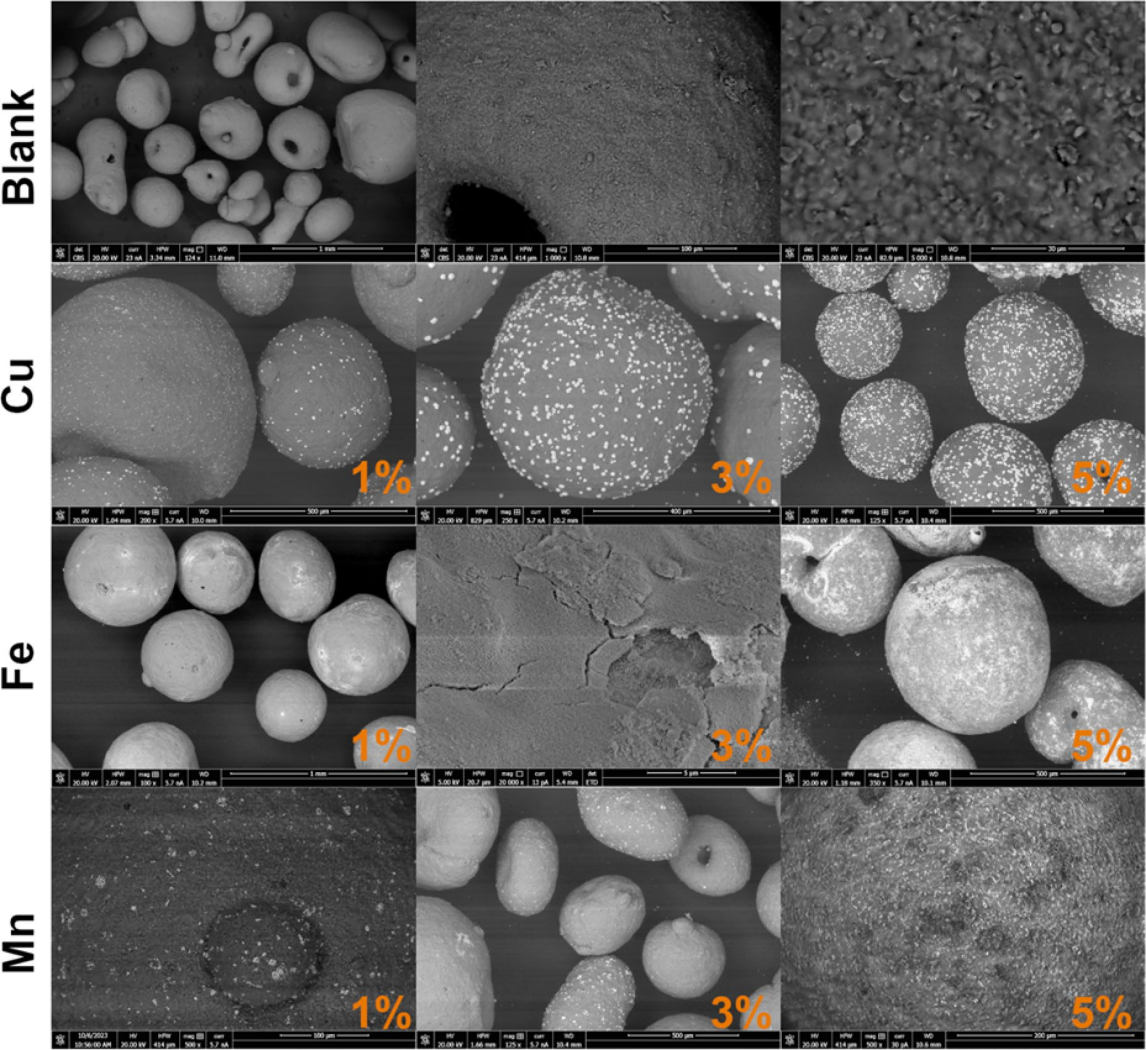
Field-emission scanning electron microscopy (FESEM) images of (**A**) unmodified cordierite (Blank, 124×, 1 000×, 5 000×), (**B**) CuO-modified cordierite (200×, 250×, 125×), (**C**) Fe₂O₃-modified cordierite (100×, 20 000×, 350×) and (**D**) MnO₂-modified cordierite (500×, 125×, 500×). These images show surface morphology changes induced by acid activation, transition-metal loading and calcination. Detailed high-resolution SEM images of all samples at additional magnifications are provided in the Supplementary Materials: 10.58032/AGH/VEVKEJ (source: own elaboration).

Figure 4 presents representative SEM micrographs of unmodified cordierite (blank) and samples modified with copper, iron, and manganese at varying concentrations (1%, 3%, and 5%). The magnifications used for each image are given in the Fig. 4 caption, and additional high-resolution images are available in Supply Materials. The unmodified cordierite showed a compact, relatively smooth surface with visible grain boundaries and no significant porosity, consistent with its C410 specification. Acid activation did not visibly increase porosity, indicating limited etching effectiveness under the selected conditions.

In contrast, modified samples exhibited clear changes in surface features. For copper-modified cordierite, small, rounded CuO particles were dispersed over the surface. SEM observations indicate that the deposited metal oxides formed surface structures ranging from approximately 1 to 10 μm. With increasing loading, CuO and Fe₂O₃ coatings became denser and more defined, suggesting partial nucleation and agglomeration during calcination, whereas MnO₂ showed a tendency toward excessive clustering. Manganese-treated samples revealed darker, irregular clusters consistent with MnO₂ formation, which appeared less uniformly distributed. At higher loadings (5%), agglomerates were larger and less integrated with the cordierite substrate, potentially reducing the specific surface area accessible to gas-phase anthracene and thereby lowering removal efficiency.

Iron-modified samples showed the most distinctive morphology, with a continuous, flaky layer covering the entire surface. This crust-like appearance indicates the formation of a uniform Fe₂O₃ film, potentially due to better wettability or reactivity of the Fe(NO₃)₃ precursor with the cordierite surface. The smoother, homogeneous coating observed in Fe-treated samples may enhance surface availability, consistent with their superior anthracene removal performance observed in later sections.

The elemental composition of the surfaces was quantified using EDS, and results are summarized in Table 2. The analysis revealed a clear correlation between precursor concentration and surface metal content. For 5% solutions, surface metal concentrations reached:

- **Cu**: 8.23%,
- **Fe**: 14.10%,
- **Mn**: 4.45% (plus residual Cl: 0.20–0.93%).

**Table 2:** Results of EDS analysis of materials. Presented values shown in mass %. The method yields results comprising elements that collectively add up to 100%. Empty fields mean that element was not detected.

	O	Na	Mg	Al	Si	Cl	K	Ca	Ti	Mn	Fe	Cu
Blank	48.17	0.23	3.82	19.87	24.43	Nd	1.99	0.19	0.35	Nd	0.96	Nd
Cu 1%	62.28	0.27	2.68	15.07	17.24	Nd	0.95	0.12	0.15	Nd	0.31	1.26
Cu 3%	59.05	Nd	2.96	15.11	16.96	Nd	0.89	0.10	0.12	Nd	0.31	4.61
Cu 5%	55.32	Nd	3.49	16.28	15.49	Nd	0.83	Nd	0.12	Nd	0.31	8.23
Fe 1%	61.09	Nd	2.69	12.87	14.37	Nd	0.95	0.14	0.14	Nd	7.87	Nd
Fe 3%	60.47	Nd	1.74	12.69	14.19	Nd	0.90	0.15	0.12	Nd	9.92	Nd
Fe 5%	60.26	Nd	1.71	11.63	11.42	Nd	0.76	Nd	0.16	Nd	14.10	Nd
Mn 1%	60.62	0.21	2.99	14.77	18.43	0.31	1.10	0.00	0.21	1.04	0.40	Nd
Mn 3%	60.88	0.19	2.41	14.81	16.70	0.93	0.97	0.12	0.15	2.64	0.33	Nd
Mn 5%	60.55	0.21	2.93	14.80	15.72	0.20	1.02	0.12	0.13	4.45	0.34	Nd

Nd – the specific element was not detected during the analysis

The unmodified blank sample showed trace amounts of Fe (~ 0.96%), consistent with its natural composition, and no detectable Cu or Mn. The presence of oxygen, silicon, aluminum, and magnesium in all samples reflects the base composition of cordierite.

In the case of manganese samples, EDS revealed residual chlorine even after calcination, confirming incomplete conversion of MnCl₂ to MnO₂ under the applied thermal conditions. This may impact long-term chemical stability and could lead to the release of HCl or other chlorine-containing species during operation. Residual chlorine detected in Mn-modified samples reflects incomplete conversion of the MnCl₂ precursor during calcination, likely due to kinetic limitations or insufficient oxidation conditions. The higher Cl content observed at 3% Mn loading compared to 5% probably reflects local heterogeneity of oxide deposition and the semi-quantitative nature of EDS analysis at low concentration levels rather than a true compositional trend.

It should be noted that EDS is a surface-sensitive technique (sampling depth: ~1–2 μm) and may not fully capture metal distribution within the bulk of the material. Furthermore, the quantification is relative and normalized to 100%, so trace elements and impurities may be underrepresented.

Overall, SEM/EDS results confirmed that:

- Metal deposition was successful,
- Surface concentration increases with precursor concentration,
- Morphological characteristics varied significantly between metal types,
- And homogeneity of metal distribution may correlate with functional performance.

These observations provide an important structural context for interpreting the anthracene removal efficiencies discussed in Sect. Critical assessment and practical implications.

### Anthracene removal efficiency

The effectiveness of transition metal-modified cordierite in removing anthracene from a gas stream was assessed under controlled thermal conditions (300 °C, 60 mL/min, 35 min). The anthracene input was fixed at 200 µg per test. The unmodified cordierite (blank) removed an average of 17.8 µg (~ 9%), which is attributed to limited physisorption at elevated temperature. This baseline value was subtracted from all modified sample results to isolate the effect of the metal oxide coatings.

The results revealed clear differences in performance depending on the type and concentration of metal:

- Copper-modified samples (CuO) showed the highest removal efficiencies among all tested materials. Removal increased with loading: 1% Cu: 14.0% efficiency (after blank correction), 3% Cu: 17.3%, 5% Cu: 25.3%, removing over 50 µg of anthracene. This trend suggests that copper oxide is catalytically active toward anthracene degradation, likely via redox cycling (Cu²⁺/Cu⁺), enabling partial oxidation of adsorbed PAHs. SEM/EDS analysis supports this interpretation, showing relatively uniform CuO distribution.
- Iron-modified samples (Fe₂O₃) also exhibited a positive dose–response relationship, though less pronounced than CuO: 1% Fe: 9.2%, 3% Fe: 13.7%, 5% Fe: 18.2%. The consistent, crust-like oxide layer observed in SEM images may have promoted more stable adsorption sites, though Fe₂O₃ is less redox-active than CuO at 300 °C. Nevertheless, iron modification proved superior to unmodified cordierite, confirming its contribution to PAH interaction.
- Manganese-modified samples (MnO₂) performed worse than expected and did not follow a linear trend with increasing loading: 1% Mn: 0%, 3% Mn: 5.6%, 5% Mn: 4.9%. The decline in efficiency despite increased loading suggests surface passivation or agglomeration of MnO₂ particles, which may have reduced the number of available active sites. Residual chlorine detected via EDS further indicates incomplete precursor decomposition and potential interference with oxidation processes.

A summary of the removal efficiencies is presented in Table 3. The superior performance of Cu- and Fe-modified samples aligns with their known catalytic behavior in oxidative environments. In contrast, MnO₂ may require different preparation conditions or precursor types to exhibit comparable activity.

**Table 3:** Amount of anthracene removed from stream containing in total 200 µg of PAH and removal efficiency of process.

Modification case	$\:{\varvec{A}}_{\varvec{r}\varvec{e}\varvec{m}\varvec{o}\varvec{v}\varvec{e}\varvec{d},\varvec{u}\varvec{n}\varvec{c}\varvec{o}\varvec{r}\varvec{r}\varvec{e}\varvec{c}\varvec{t}\varvec{e}\varvec{d}}$ $\:\left[\varvec{\mu\:}\varvec{g}\right]$	$\:{\varvec{A}}_{\varvec{r}\varvec{e}\varvec{m}\varvec{o}\varvec{v}\varvec{e}\varvec{d},\varvec{n}\varvec{e}\varvec{t}}$ $\:\left[\varvec{\mu\:}\varvec{g}\right]$	$\:\varvec{\upeta\:}\:\left[\mathbf{\%}\right]$
Blank	17.8	-	-
Cu 1%	45.8	28.0	14.0
Cu 3%	52.3	34.5	17.3
Cu 5%	68.3	50.5	25.3
Fe 1%	36.2	18.4	9.2
Fe 3%	45.2	27.4	13.7
Fe 5%	54.1	36.3	18.2
Mn 1%	17.6	0.0	0.0
Mn 3%	29.1	11.3	5.6
Mn 5%	27.6	9.8	4.9

The results confirm that transition metal modification enhances the functional properties of cordierite toward anthracene removal, with copper-based formulations showing the highest potential under the tested conditions. The slightly lower removal observed for Mn 1% compared to the blank falls within measurement uncertainty and may also reflect partial blocking of native cordierite catalytic sites by non-uniform MnO₂ deposition at low loading, as indicated by the EDS results.

### Critical assessment and practical implications

The observed anthracene removal efficiencies indicate that transition metal modification significantly enhances the sorptive and potentially catalytic properties of cordierite, particularly when coated with copper or iron oxides. Among the tested oxides, CuO demonstrated the highest removal performance, followed by Fe₂O₃. MnO₂-modified samples showed unexpectedly poor results, which may be related to precursor selection, oxide dispersion, or surface poisoning effects.

**Fig. 5:**
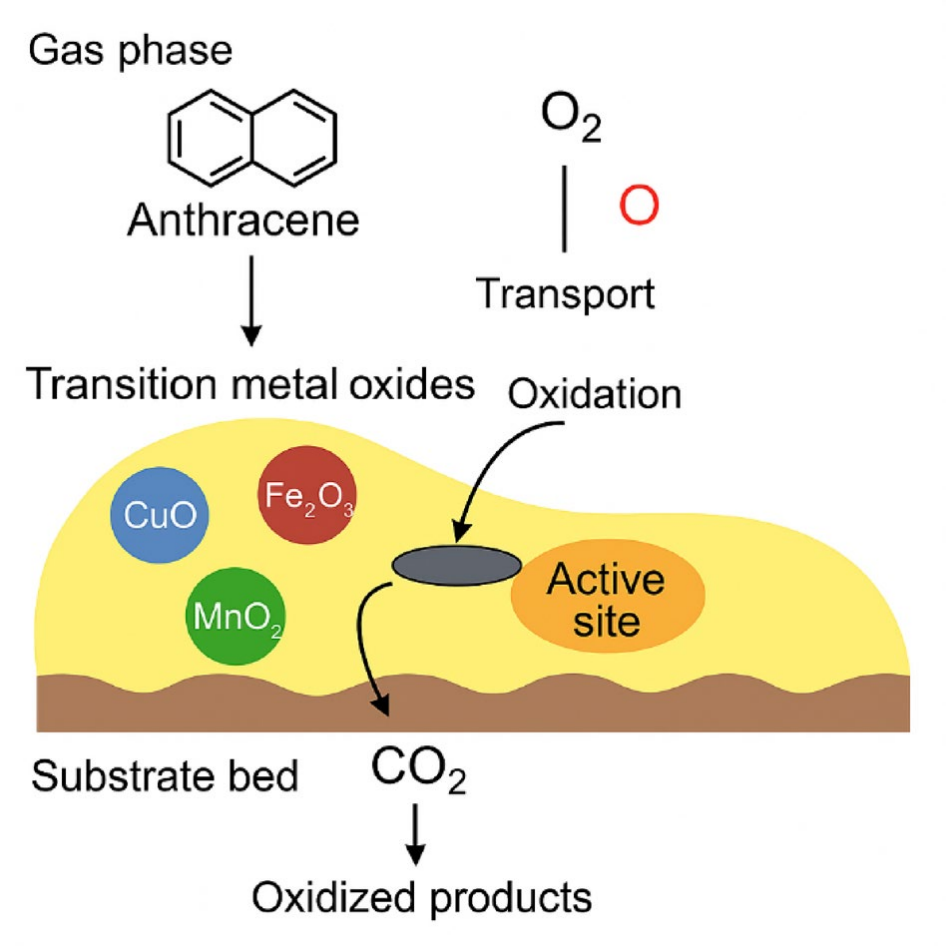
Proposed mechanism of anthracene removal on transition metal oxides (CuO, Fe₂O₃, MnO₂). Anthracene from the gas phase is adsorbed onto active oxide surfaces, where it undergoes oxidative degradation in the presence of activated O₂. Redox cycling (e.g., Cu²⁺/Cu⁺) is critical to catalytic function. Surface defects and oxide dispersion modulate the effectiveness of the process. (source: own elaboration).

To explain these differences, a conceptual mechanism of anthracene removal was proposed and is illustrated in Fig. 5. The process is likely initiated by the physisorption or weak chemisorption of anthracene molecules onto the metal oxide surface. This interaction is facilitated by π–d orbital overlap between the aromatic system and surface metal cations (Cu²⁺, Fe³⁺, Mn⁴⁺). Subsequently, surface-bound or gas-phase molecular oxygen (O₂) is activated by the oxide lattice, leading to partial oxidation of anthracene into CO₂ and less harmful intermediates. Redox-active centers such as Cu²⁺/Cu⁺ and Fe³⁺/Fe²⁺ facilitate electron transfer, enhancing oxidative degradation. The proposed mechanism of oxygen activation resembles dissociative chemisorption followed by spillover, in which O₂ molecules adsorb on redox-active metal sites, dissociate into atomic oxygen, and migrate across the surface to participate in anthracene oxidation. This mechanism has been widely reported for transition-metal oxide catalysts^31,32^ and is consistent with the activity trends observed here.

This proposed mechanism also aligns with the morphological findings discussed in Sect. 3.2. In particular:

- Copper formed finely dispersed particles, increasing accessible active surface area.
- Iron formed continuous, crust-like layers with homogeneous distribution.
- Manganese formed dense, non-uniform agglomerates with residual Cl⁻, likely reducing catalytic effectiveness.

The observed anthracene removal performance of CuO- and Fe₂O₃-modified cordierite materials under simulated gasoline exhaust conditions reflects the catalytic potential of non-noble metal oxides in real-world environmental applications. These findings are in line with recent developments in environmental catalysis that emphasize low-cost, redox-active materials for VOC and PAH mitigation^33^.

Comparatively, previous studies have demonstrated high catalytic oxidation efficiency of PAHs using metal oxides and mixed-metal systems, particularly in aqueous or high-temperature gas-phase environments. For example^34^, achieved selective oxidation of anthracene to anthraquinone at 400–450 °C using vanadium- and molybdenum-based catalysts, showing that transition metals can facilitate ring-opening and oxygen insertion under thermal conditions. Our approach differs in using air as the oxidant and omitting NO_x_ or ClO₂ additives, focusing instead on the potential for passive control strategies embedded in exhaust filtration.

Studies by^35^ and^22^ confirm the synergistic effects of CuO and Fe-based materials in anthracene degradation via oxidation, both in soils and aqueous media. Our gas-phase setup expands on this by demonstrating comparable oxidation capacity at 300 °C without solvents or liquid-phase catalysts, supporting the feasibility of ceramic-supported oxides in dry emission contexts.

A key mechanistic insight is supported by the work of^36^, who highlighted the Mars–van Krevelen mechanism as central to PAH total oxidation on transition metal oxides, especially under oxygen-rich conditions. Our results are consistent with this mechanism, suggesting that lattice oxygen from metal oxides participates in surface oxidation of anthracene, followed by regeneration through gaseous O₂.

However, the relatively poor performance of MnO₂-modified cordierite aligns with recent findings by Sui et al.^37^, indicating that unsupported MnOₓ catalysts may suffer from lower stability, limited oxygen mobility, and tendency toward particle agglomeration under thermal stress. This underlines the importance of oxide dispersion and redox tailoring in catalyst design.

Furthermore, recent advances in catalyst engineering point to the role of surface oxygen vacancies and defect states in enhancing catalytic reactivity, particularly in Cu-based systems. Ye et al., 2022^38^ demonstrated the generation of environmentally persistent free radicals (EPFRs) on Cu-containing frameworks, which are hypothesized to contribute to enhanced adsorption and surface-initiated reactions. Although EPFR quantification was beyond the scope of our study, the elevated anthracene removal for CuO-modified samples may suggest similar surface radical involvement.

Finally, it is important to note the gap between lab-scale performance and real-world applicability. As emphasized by Dai^33^, integrated pollution control systems must account for multi-component gas streams, fluctuating temperatures, and long-term durability. Our simplified setup serves as a controlled testbed, but future studies should address mixed-PHA matrices, aging effects, and regeneration cycles under cyclic exhaust conditions.

Together, these comparisons highlight that while noble metal-free oxides may not yet rival platinum-group catalysts in absolute efficiency, their selectivity, availability, and cost-efficiency render them promising candidates for supplemental or distributed emission control strategies in vehicular and stationary sources.

Despite promising trends, several practical limitations of the current approach must be acknowledged:

- The experiments were conducted at 300 °C and 60 mL/min flow rate – conditions that do not directly replicate real exhaust dynamics in gasoline vehicles, where higher flow rates (in L/min or m³/h range) and transient temperature profiles dominate. Under such conditions, the residence time of exhaust gases is much shorter, which may significantly reduce PAH interaction with the catalyst surface.
- The use of nitrate (Cu(NO₃)₂, Fe(NO₃)₃) and chloride (MnCl₂) salts introduces concerns about NOx and HCl release during calcination and potential residual contamination of the catalyst. Future studies should explore alternative precursors such as oxalates, acetates, or organometallic complexes to mitigate this issue.
- Especially in the case of CuO, previous studies have suggested its involvement in the formation of environmentally persistent free radicals (EPFRs) during incomplete combustion or degradation of aromatic compounds. While this was not evaluated in the current study, it warrants caution and further analysis, especially for real-world implementation.
- In practical applications, gasoline particulate filters are either uncoated (bare cordierite) or washcoated with PGMs. Adding a separate layer of transition metal oxides could face engineering barriers, such as compatibility with existing washcoat formulations or risk of blockage. Moreover, applying these materials in a way that preserves gas–solid contact is non-trivial and may require redesigning the filter geometry.

Despite these limitations, the results demonstrate that non-PGM catalytic materials based on Cu and Fe oxides have potential for supplementary control of semi-volatile organic pollutants such as PAHs. This aligns with growing efforts to reduce the reliance on critical raw materials and integrate more sustainable solutions into emission control systems.

## Conclusions

This study demonstrates that transition-metal modification of cordierite can significantly enhance its capacity to capture and partially oxidize a representative PAH (anthracene) under gasoline exhaust-like conditions. Through combined material synthesis, thermal and surface characterization, and direct gas-phase testing, clear structure–activity relationships were established between oxide dispersion, morphology and functional performance. Copper- and iron-based coatings emerged as the most promising, reflecting the importance of redox-active surfaces and uniform coverage, while manganese coatings highlighted limitations related to precursor selection and oxide agglomeration. Beyond confirming the feasibility of low-cost alternatives to platinum-group catalysts, the findings provide mechanistic insight into the interaction of transition-metal oxides with PAHs – via adsorption and oxygen-mediated oxidation – and offer guidance for optimizing coating composition and processing. Such insights may inform the design of next-generation gasoline particulate filters or supplementary catalytic layers aimed at mitigating unregulated micro-contaminants. Future research should address dynamic exhaust conditions, explore mixed-metal and hierarchical coatings, test alternative precursors to minimize residual anions, and evaluate long-term stability and regeneration. Advancing these directions could accelerate the development of sustainable emission-control strategies that reduce dependence on critical raw materials while improving air quality.

## Data Availability

Supplies materials are available at: Pacura, W.; Górecki, J.; Vanhaecke, E.M.M.; Szramowiat-Sala, K.; Gierek, M.; Gołaś, J. Supplementary materials for: Selected Micro-contaminants Removal by Cordierite Modified with Transition Metals. https://doi.org/10.58032/AGH/VEVKEJThe materials contain following positions: cordierite certificate; full scale SEM figures; SEM figures of incipient wetness impregnation; alternative methods of anthracene trapping mechanism.Any other information is duly available from the first author on reasonable request.
